# Ivabradine: An Intelligent Drug for the Treatment of Ischemic Heart Disease

**DOI:** 10.3390/molecules171113592

**Published:** 2012-11-16

**Authors:** Graziano Riccioni

**Affiliations:** Intensive Cardiology Care Unit, San Camillo de Lellis Hospital, Manfredonia, via Isonzo, 71043 Foggia, Italy; Email: griccioni@hotmail.com; Tel.: +39-882-227-022

**Keywords:** angina, coronary artery disease, fanny current, HCN channels, heart rate, ivabradine, sino atrial node

## Abstract

Heart rate (HR) is a precisely regulated variable, which plays a critical role in health and disease. Elevated resting HR is a significant predictor of all-cause and cardiovascular mortality in the general population and patients with cardiovascular disease (CVD). β-blocking drugs exert negative effects on regional myocardial blood flow and function when HR reduction is eliminated by atrial pacing; calcium channel antagonists (CCAs) functionally antagonize coronary vasoconstriction mediated through α-adreno-receptors and are thus devoid of this undesired effect, but the compounds are nevertheless negative inotropes. From these observations derives the necessity to find alternative, more selective drugs to reduce HR through inhibition of specific electrical current (I*_f_*). Ivabradine (IVA) is a novel specific HR-lowering agent that acts in sinus atrial node (SAN) cells by selectively inhibiting the pacemaker I*_f_* current in a dose-dependent manner by slowing the diastolic depolarization slope of SAN cells, and by reducing HR at rest during exercise in humans. Coronary artery diseases (CAD) represent the most common cause of death in middle–aged and older adults in European Countries. Most ischemic episodes are triggered by an increase in HR, that induces an imbalance between myocardial oxygen delivery and consumption. IVA, a selective and specific inhibitor of the I*_f_* current which reduced HR without adverse hemodynamic effects, has clearly and unequivocally demonstrated its efficacy in the treatment of chronic stable angina pectoris (CSAP) and myocardial ischemia with optimal tolerability profile due to selective interaction with I*_f_* channels. The aim of this review is to point out the usefulness of IVA in the treatment of ischemic heart disease.

## List of Abbreviations

CSAP: Chronic stable angina pectoris
HR: Heart rate
BP: Blood pressure
CBF: Coronary blood flow
CVD: Cardiovascular disease
CAD: Coronary artery disease
SAN: Sino atrial node
LVEF: Left ventricular ejection fraction
CHF: Chronic heart failure
IVA: Ivabradine

## 1. Introduction

Angina is a chest discomfort caused by myocardial ischemia without necrosis, and is further qualified by its precipitating factors, time course to relief, and clinical characteristics, such as radiation and quality. Typical angina may be triggered by increased activity, emotional stress, cold, wind, and fever [1]. Chronic stable angina pectoris (CSAP) is generally due to one or more significant obstructive lesions in the coronary arteries, obstructive lesions defined as stenosis of 50% of the diameter of the left main coronary artery or stenosis of 70% of the diameter of a major epicardial vessels. Precipitating circumstances remain similar between episodes, thresholds may be predicted by patients, and relief patterns become known. Since stenoses are fixed, the angina is due to demand ischemia [2].

A primary factor in CSAP results from myocardial ischemia, which is caused by an imbalance between myocardial O_2_ requirements and myocardial O_2_ supply [1]. Usually this is simply referred to as an imbalance between myocardial oxygen supply and demand, but it should be clear that substrate supply, utilization, and enzymatic activities, along with other variables involved in intermediary metabolism and mitochondrial function, also play a major role in the pathogenesis of myocardial ischemia in angina, acute coronary syndromes, and during reperfusion ischemic injury [3].

Major determinants of myocardial oxygen demand are heart rate (HR), contractility, blood pressure (BP), and systolic wall stress, in turn influenced by preload, afterload, and contractility. Since myocardial oxygen extraction from coronary arterial blood at rest is normally high, about 75% of arterial oxygen content, adjustments in oxygen extraction cannot correct an imbalance. Physiological increases in myocardial oxygen needs are normally provided by rises in coronary blood flow (CBF) [1].

Other factors involved in pathophysiology of angina are represented by alteration of coronary vasomotor control and endothelial function. In particular defects in endothelium-dependent dilation in atherosclerotic epicardial coronary arteries that vasoconstrict in response to stimuli that normally cause vasodilation, such as acetylcholine, exercise, or cold pressure testing. Pathological vasomotor control was found in CSAP patients with angiographically normal coronary arteries in which the chest pain is due to a reduction of endothelium-dependent vasodilation of resistance arteries. The same defect is present in patients with left ventricular hypertrophy associated with hypertension, another condition that may be associated with angina pectoris with normal coronary angiography [2].

Increasing evidence suggest that CSAP may be caused by transient reductions in O_2_ due to coronary vasoconstriction mediated through α-adrenoreceptors, by dynamic changes in smooth muscle tone and also to constriction of arteries distal to the stenosis [1].

In patients with CSAP, a fixed reduction in the diameter of coronary arteries by at least 70% dictates an obligatory reduction in CBF in one or more coronary arteries. The inability to increase oxygen extraction or CBF, together with elevated myocardial energy demand, leads to anginal pain, variably accompanied by panoply of metabolic, electrophysiologic, and hemodynamic consequences. Most events that trigger angina do so by changing myocardial oxygen demand, increases in HR, afterload, preload, or contractility. Similarly, the beneficial effects of most maneuvers that relieve angina may be explained through corrective alterations in the determinants of myocardial oxygen supply and demand [3].

CSAP is often the first manifestation of ischemic heart disease. The effective management of this highly prevalent condition is largely dependent on the identification of the prevailing pathogenic mechanism, the implementation of lifestyle changes, the appropriate use of pharmacological agents, and revascularization techniques. The treatment of CSAP has improved in recent years as a result of a better understanding of its pathogenic mechanisms. Understanding the pathogenesis of the disease is important to identify effective treatment strategies. A careful clinical history, the implementation of appropriate diagnostic tests and a rational use of anti-anginal drugs often ensure the successful control of the patient's symptoms [4].

## 2. Generation of Electrical Impulse and Role of I*_f_* Current

The *pacemaker cells* are the first cells which generate the impulse that then spread to the other zone of the cardiac chambers. These cells have the peculiar feature of spontaneous depolarization, due to the ionic currents movement across specialized channels [5]. Hyperpolarization-activated cyclic nucleotide-gated (HCN) channels have a key role in the control of HR and neuronal excitability. HCN channels are unique among vertebrate voltage-gated ion channels, in that they have a reverse voltage-dependence that leads to activation upon hyperpolarization [6]. In addition, voltage-dependent opening of these channels is directly regulated by the binding of cAMP [7].

HCN channels, molecular substrates of native funny (f-) channels of cardiac pacemaker cells, are encoded by four genes (HCN1-4) and are widely expressed throughout the heart and the central nervous system [6]. The current flowing through HCN channels, designated *I*(h) or *I*(f), plays a key role in the control of cardiac and neuronal rhythmicity (pacemaker current) [8].

Among the different currents at basis of mechanisms contributing to electrical stimulus, I*_f_* current has a major role in providing pacemaking competence [9,10]. Originally described in the sino atrial node (SAN) the *funny* current and its properties and function in cardiac *pacemaker cells* have been the object of intense investigation [11]. *Funny* (f) channels underlie the cardiac pacemaker I*_f_* current, originally described as an inward current activated on hyperpolarization to the diastolic range of voltages in SAN myocytes [12,13]. The involvement of *funny* channels in the generation and modulation of cardiac pacemaker activity has been amply demonstrated by thorough analysis since its discovery [14].

The f-channel controls the rate of spontaneous depolarization of cardiac pacemaker cells. Its function is influenced by the concentration of cyclic AMP (cAMP) proximate to the channel. cAMP production in the SAN is increased by adrenergic stimulation and decreased by cholinergic stimulation [15]. Channels openings facilitated by the binding of cAMP with a consequent movement of sodium and potassium which carry the *I*_f_ current, which directly modulates the rate of spontaneous diastolic depolarization [16].

*I*_f_ current is important in the generation of pacemaking not only for diastolic-depolarization but also for its involvement in neurotransmitter-induced control of cardiac rate. It was shown since its first description that I*_f_* mediates the acceleratory effect of adrenaline o pacemaker rate [17] and later study showed its strong modulation by acetylcholine but with opposite action regard that of catecholamines [18,19].

## 3. Pharmacokinetics and Pharmacodynamics of Ivabradine

### 3.1. Chemical Structure

**Figure 1:**
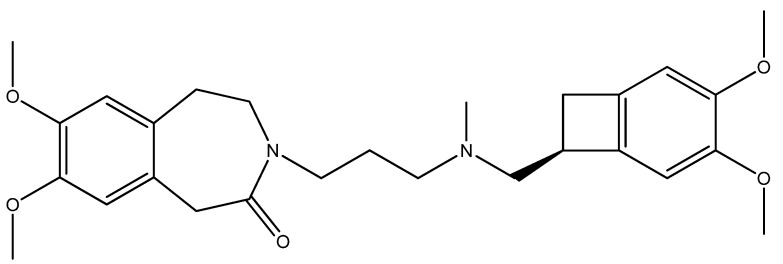
Structure of ivabradine.

Ivabradine (IVA, Procoralan^®^, Corlentor^®^, Ivabid^®^, 3-(3-{[((7*S*)-3,4-dimethoxybicyclo[4,2,0]octa-1,3,5-trien-7-yl)methyl]methylamino}propyl)-1,3,4,5-tetrahydro-7,8-dimethoxy-2H-3-benzazepin-2-one hydrochloride, Figure 1) is a specific HR lowering agent that acts in SAN cells by selectively inhibiting the pacemaker *I*_f_ in a dose-dependent manner by slowing the diastolic depolarization slope of SAN cells, and reducing HR at rest during exercise in animals and humans [20,21,22]. The amplitude of *I*_f_ current, one of the most important ionic currents regulating pacemaker activity in the SAN through a mixed Na^+^-K^+^ inward current activated by hyperpolarization, determines the slope of the diastolic depolarization phase and thereby the HR. The molecular basis of *I*_f_ and its related equivalent in non-cardiac cells *I*_f_ have been characterized by cloning a family of ionic channels, known as HCN, which stands for for hyperpolarization activated cyclic nucleotide-gated channels (HCN) [17,23].

Detailed patch-clamp studies in rabbit SAN cells have shown that IVA blocks *I_f_* channels in a use-dependent way and that it interacts with the channels from the intracellular side [24]. More recently, also in SAN cells, IVA has been shown exclusively to be an open channel blocker, indicating that it cannot reach its binding site when the channels are closed, and its blocking effect is current-dependent and is attenuated during very long hyperpolarized pulses (more than 20 s of hyperpolarization) [25]. At therapeutic concentrations, IVA has no effect on other cardiac ion channels, and it does not act via changing cAMP levels in cardiac cells. IVA, unlike conventional HR-lowering agents including non-dihydropyridine calcium channel blockers and β-blockers, has no direct effects on myocardial contractility, ventricular repolarization, intracardiac conduction, and improve left ventricular ejection fraction (LVEF) in patients with chronic heart failure and cardiac systolic dysfunction [26].

### 3.2. Activity

The pharmacokinetics and pharmacodynamic features of IVA have been studied in experiments with animals, cellular cultures, and in healthy volunteers. Animal model studies in SAN cells have shown that *I*_f_-channel binding/unbinding IVA are restricted to the open channel state, implying that IVA is an *open channel blocker* [24]. A peculiar feature of IVA is that its blocking action is not intrinsically voltage-dependent, but rather depends on the direction of ion flow across the channel pore [27].

In an important study with the aim to analyse the plasma concentration-bradycardic effect relationships after administration of IVA under different condition (routes of administration, type of dosing, different levels of effects) several important aspects of pharmacokinetic and pharmacodynamic of IVA have shown. The maximal plasma concentrations of IVA were observed between 1 and 1.5 h after the oral administrations (single or repeated). Plasma concentrations of S-18982, the *N*-dealkylated metabolite, peaked between 1 and 1.5 h after the oral administrations and at 1 hour after intravenous bolus. The comparison of the area under the curve (AUC) values obtained after single and repeated administrations of IVA showed that there was no accumulation of the parent compound. The AUC of S-18982 increased significantly between the first dose and after 4 days of administration of 10 mg dose and 20 mg dose. The metabolite/drug AUC ratios for the 10 mg oral dose were 32.3% ± 5.0% after the single administration and 41.4% ± 9.0% after the repeated administration; corresponding values for the 20 mg dose were 32.5% ± 5.2% and 43.9% ± 9.2%. There was no statistically significant difference between the AUC of the 10 mg doses and AUC/2 of the 20 mg doses for either S-16257 or S-18982 during both single and repeated administration. No significant change in the duration of PR and QRS intervals at rest was found after IVA administration. Regarding to HR at rest the difference between single 10 mg oral doses or 20 mg dose and the same dosage bid repeated doses was statistically significant for repeated administration. With respect to exercise HR the maximal effects of the 20 mg dose were significantly greater than the maximal effects of the 10 mg dose for both single and repeated administration. In the conclusion this study confirms the HR lowering effect of IVA in healthy volunteers after single intravenous administration (10 mg) and single and repeated oral administration (10 and 20 mg bid), with a dose-dependent effect [23].

### 3.3. Relationship

Since 1980 several drugs, originally named *Pure Bradycardic Agents* with the ability of depressing diastolic depolarization rate, have been developed. They were shown to be f*-channel blockers* [28,29]. The first such drug was *alinidine*, an N-allyl derivative of clonidine [30], followed by other molecules with anti-anginal effects improving the relation between specificity of inhibitions and side effects such as *falipalim* and its congener UL-FS49 [29], and ZD7288 [31]. IVA is the only compound with this mechanism of action presently available for clinical use.

## 4. Safety and Tolerability

IVA has been associated with a good safety profile during its clinical development, postmarketing surveillance, and ongoing clinical trials in adult and elderly patients [32,33,34,35].

### 4.1. Phosphenes

IVA can interact with the retinal current *I*_h_, which participates in the temporal resolution of the visual system, by curtailing the retinal response to bright light stimuli. Visual side effect symptoms known as luminous phenomena or phosphenes represent the most common adverse effects, are mild and transient, and not affecting patients’ ability to carry out normal activities. The incidence of phosphenes in clinical trials was markedly lower (≤3%), than in previous studies, in which it was over reported due to special questions about visual symptoms [36].

The interaction of IVA with the visual system by inhibiting hyperpolarization-activated current in retinal cells (*I*_h_) with properties similar to cardiac *I*_f_ has been reported in retinal neurones. *I*_h_ inhibition, by altering at the retinal synapses the filtering of signals generated by thermal breakdown of rhodopsin or other fluctuations, is expected to increase the probability of phosphenes occurrence [37]. They are described as a transient enhanced brightness in a limited area of the visual field and are triggered by sudden changes in light intensity. The onset occurs in the first two months of treatment after which they may occur repeatedly; all phosphenes resolved during or after treatment and there is no evidence that IVA affects driving performance or the ability to operate machinery [32,38]. IVA does not cross the blood-brain barrier and therefore, has no effect on the I*_h_* current in central nervous system neurons and other tissues [35].

### 4.2. Bradycardia and Conduction/Rhythm Disturbances

Unlike many rate-lowering agents, IVA reduces HR in a dose-dependent manner both at rest and during exercise. The uncommon bradycardic effect of IVA is proportional to the resting HR, such that the effect tends to plateau. Thus, extreme sinus bradycardia is uncommon, even in octogenarians patients with increased incidence of bradycardia due to age-related alteration of the sinus node [33]. Less than 1% of patients withdrew from therapy because of untoward sinus bradycardia [35]. The BEAUTIFUL Holter substudy explored the cardiac safety of the *I*_f_ inhibitor IVA in patients with stable CAD and left ventricular systolic dysfunction receiving optimal background therapy. This study has demonstrated no increase in incidence of conduction and rhythm disturbances, and confirmed that IVA significantly lowers HR without raising concern for cardiac safety even in combination with β-blockers therapy [39,40]. IVA is safe in reducing HR in patients referred for computed tomography coronary angiography [41], with inappropriate sinus tachycardia (IST) [42], diabetes mellitus [43], and after cardiac transplantation [44].

### 4.3. QT Interval

QT interval is expectedly prolonged with the reduction in HR, but after appropriate correction for HR and in direct comparisons of the QT interval when the influence of the HR was controlled by atrial pacing, no significant effect of IVA on ventricular repolarization duration was demonstrated [32]. Consequently, IVA has no direct torsadogenic potential, although, for obvious reasons, the specific bradycardic drug should not be administered with agents which have known QT prolonging effects [45]. Even animal studies have demonstrated that IVA dose-dependently induced bradycardia without altering QT [46,47].

### 4.4. Interactions with Other Drugs

The concomitant use of potent CYP3A4 inhibitors such as azole antifungals (ketoconazole, itraconazole), macrolide antibiotics (clarithromycin, erythromycin *per os*, josamycin, telithromycin), HIV protease inhibitors (nelfinavir, ritonavir) and nefazodone is contra-indicated. The potent CYP3A4 inhibitors ketoconazole (200 mg once daily) and josamycin (1 g twice daily) increased IVA mean plasma exposure by 7- to 8-fold. Specific interaction studies in healthy volunteers and patients have shown that the combination of IVA with the HR reducing agents diltiazem or verapamil resulted in an increase in IVA exposure (2 to 3 fold increase in AUC) and an additional HR reduction of 5 bpm [48]. The concomitant use of IVA with these medicinal products is not recommended. The concomitant use of IVA with other moderate CYP3A4 inhibitors (e.g., fluconazole) may be considered at the starting dose of 2.5 mg twice daily and if resting HR is above 60 bpm, with monitoring of HR. IVA exposure was increased by 2-fold following the co-administration with grapefruit juice. Therefore the intake of grapefruit juice should be restricted during the treatment with IVA [49,50]. CYP3A4 inducers (e.g., rifampicin, barbiturates, phenytoin, *Hypericum perforatum* [St John’s Wort]) may decrease IVA exposure and activity. The concomitant use of CYP3A4 inducing medicinal products may require an adjustment of the dose of IVA. The combination of IVA 10 mg twice daily with St John’s Wort was shown to reduce IVA AUC by half. The intake of St John’s Wort should be restricted during the treatment with IVA. Carbamazepine interacts clinically significant with IVA in healthy volunteers, and lowers its bioavailability by about 80% [48,50].

## 5. Antianginal and Antischemic Effects

Studies in healthy and asymptomatic subjects as well as in patients with already established CAD have demonstrated that HR is very important and major independent cardiovascular risk for prognosis [51]. Epidemiologic and long-term follow-up studies have demonstrated an independent association between HR and cardiovascular mortality, CAD, and sudden cardiac death in healthy subjects [52]. In patients with CAD, elevated HR is an independent risk predictor for major ischemic coronary events, cardiovascular mortality, and sudden cardiac death [53]. In patients with CHF, baseline HR is an independent risk factor of all-cause mortality, cardiovascular mortality, and hospitalization for CHF. HR is a major determinant of myocardial oxygen consumption and energy utilization; furthermore, an increase in HR reduces the diastolic coronary perfusion time. An increase in HR as a consequence of increased sympathetic activity may trigger ischemic events [54].

IVA is the first of a new class of bradycardic agents without other direct cardiovascular effects (negative inotropic effect, blood pressure reduction) [55,56] which can be safely combined with other currently used cardiovascular drugs (β-blockers and calcium channel antagonists) for reducing HR [57].

IVA at doses of 5.0, 7.5 and 10.0 mg/bid has demonstrated a non-inferior anti-anginal and anti-ischemic effects respect to atenolol (50 or 100 mg/day) in 939 patients with CSAP [58]. In this study Tardif and coll. [59] found that the increase in exercise capacity was associated with a prolongation of exercise test duration. Even in combination with atenolol IVA has demonstrated in 889 patients with CSAP a significant increase in total exercise duration (primary efficacy criterion), and improvement in other exercise test criteria (time to limiting angina, time to angina onset, and time to 1 mm ST-segment depression) respect to placebo group. This study has demonstrated that IVA can be added to β-blockers in CSAP patients with insufficient HR reduction or in whom high doses of β-blockers cannot be used because of known side effects [60]. Even in combination with low dose of bisoprolol (10 mg) IVA produces additional anti-anginal and anti-ischemic benefits, and improves chronotropic reserve in patients with stable angina [61]. In daily clinical practice, combining IVA with beta-blocker not only reduces HR, number of angina attacks, and nitrate consumption, but also improves the quality of life in patients with CSAP both in combination with β-blockers [62,63].

The morBidity–mortality EvAlUaTion of the I*_f_* inhibitor ivabradine in patients with coronary disease and left ventricULar dysfunction (BEAUTIFUL) study has demonstrated that in prespecified subgroup of patients with HR of 70 bpm or more (N = 5392), the treatment with IVA was associated with a 36% reduction in relative risk for fatal and non-fatal acute myocardial infarction (AMI) (*p* = 0.001), a 30% reduction for the need for coronary revascularization (*p* = 0.016), and a 22% reduction in the hospitalization for fatal and non-fatal AMI or unstable angina (*p* = 0.023) [63]. This study also offered a unique opportunity to evaluate prospectively for the first time the effect of HR as a prognostic factor by analyzing the effect of elevated HR on cardiovascular events in the placebo arm in this high-risk population of patients with CAD or LVD [53].

The Systolic Heart Failure Treatment with the I(f) Inhibitor Ivabradine Trial (SHIFT), a randomized, double-blind study designed to compare IVA (titrated to a maximum of 7.5 mg twice daily or matching placebo) on outcomes in 6,500 patients with symptomatic chronic heart failure (CHF) (NYHA class II-IV) and LVEF < 35%, has demonstrated the importance of HR reduction with IVA for improvement of clinical outcomes in heart failure and has confirmed the important role of HR in the pathophysiology of this disorder [36].

## 6. HR Control in Myocardial Infarction and Cardiogenic Shock

IVA is presently not indicated in the treatment of AMI and cardiogenic shock. Some studies are published in the literature which investigated the future potentiality of this drug in these two conditions. Fasullo *et al.* [64] have investigated the feasibility, tolerability, and the effects after 30 days of follow–up of IVA *versus* metoprolol in early phases of anterior ST elevation myocardial infarction (STEMI) reperfused by percutaneous coronary intervention (PCI). In this study 155 patients with a first anterior STEMI, Killip class I–II, an acceptable echocardiographic window, and with an ejection fraction (EF) < 50% 12 h after PCI were randomized to receive metoprolol (76 patients) or IVA (79 patients). The HR was significantly reduced in both groups, but IVA group showed a significant increase in EF. IVA may be administered early (12 h after PCI) to patients with successful PCI for anterior STEMI with an impaired left ventricular function and high HR and sinus rhythm.

The major cause of in-hospital AMI mortality remains myocardial failure with consecutive cardiogenic shock and multiorgan failure (MOF). Reduction of HR is one of the most important energy-saving maneuvers, which can be achieved by administration of beta-receptor-blocking agents [65]. Ongoing trial [MODI(f)Y trial] has been initiate in critically ill patients with multiple organ dysfunction syndrome (MODS). In this prospective, single center, open label randomized trial the authors will investigate the potential of IVA to reduce an elevated resting HR in MODS patients with contraindications to beta-blockers therapy. In patients with clinical signs of hypotension, however, the guidelines recommend to stabilize the patient before administering an oral beta-receptor blocker, mainly because of the hypotensive effects of the substance class. In this situation, selective heart rate reduction, e.g., via administration of ivabradine without side effects of hypotension may be advantageous and better tolerated in patients with cardiogenic shock [66].

IVA has proved to be of benefit in experimental models with the end points of ischemic myocardial blood flow and contractile function, infarct size, post-infarct remodeling and atherosclerosis. The benefits to ischemic myocardial blood flow and contractile function are strictly heart rate dependent; those on infarct size are partly heart rate independent [67,68].

## 7. Conclusions

HR is a major determinant of myocardial oxygen demand and supply, and increased HR adversely affects the pathophysiology of myocardial ischemia. High resting HR is a risk factor in CVD. The development of the HR lowering agent IVA showed that HR was also an important treatment target, notably in CAD.

Indeed, HR reduction with IVA, a selective and specific I(f) inhibitor, reduces myocardial oxygen demand, increases diastolic perfusion time and improves energetics in ischemic myocardium. IVA protects the myocardium during ischemia and reduces remodeling following myocardial infarction. It improves prognosis in patients with CAD, left ventricular dysfunction and HR ≥ 70 beats per minute, as well as in patients with heart failure and left ventricular dysfunction. IVA is selective, safe, well tolerated and can be used in combination with the main drugs for CVD [68,69,70].
